# Expression patterns of HNF4α, TTF-1, and SMARCA4 in lung adenocarcinomas: impacts on clinicopathological and genetic features

**DOI:** 10.1007/s00428-024-03816-6

**Published:** 2024-05-06

**Authors:** Hitomi Kawai, Tamaki Miura, Natsumi Kawamatsu, Tomoki Nakagawa, Aya Shiba-Ishii, Taichiro Yoshimoto, Yusuke Amano, Atsushi Kihara, Yuji Sakuma, Kazutaka Fujita, Tomoki Shibano, Shumpei Ishikawa, Tetsuo Ushiku, Masashi Fukayama, Hiroyoshi Tsubochi, Shunsuke Endo, Koichi Hagiwara, Daisuke Matsubara, Toshiro Niki

**Affiliations:** 1 Department of Pathology, University of Tsukuba, 1-1-1 Tennodai, Tsukuba, Ibaraki 305-8574 Japan; 2 Department of Diagnostic Pathology, University of Tsukuba Hospital, 2-1-1 Amakubo, Tsukuba, Ibaraki 305-8576 Japan; 3 Department of Integrative Pathology, Jichi Medical University, 3311-1 Yakushiji, Shimotsuke, Tochigi 329-0498 Japan; 4 Department of Pathology, Showa General Hospital, 8-1-1 Hanakoganei, Kodaira-Shi, Tokyo 187-851 Japan; 5 Department of Molecular Medicine, Sapporo Medical University, 1-17, Minami Chuo-Ku, Sapporo, Hokkaido 060-8556 Japan; 6 Department of Respiratory Medicine, Jichi Medical University, 3311-1 Yakushiji, Shimotsukeshi, Tochigi 329-0498 Japan; 7 Department of Thoracic Surgery, Jichi Medical University, 3311-1 Yakushiji, Shimotsukeshi, Tochigi 329-0498 Japan; 8 Department of Preventive Medicine, Graduate School of Medicine, the University of Tokyo, 7-3-1 Hongo, Bunkyo-Ku, Tokyo 113-0033 Japan; 9 Human Pathology Department, Graduate School of Medicine, the University of Tokyo, 7-3-1 Hongo, Bunkyo-Ku, Tokyo 113-0033 Japan; 10 Omiya Medical Association Medical Examination Center, 2-107, Higashioonari-Chou, Kita-Ku, Saitama-Shi, Saitama, 331-8689 Japan

**Keywords:** Lung adenocarcinoma, HNF4α, TTF-1, SMARCA4, KRAS, A549

## Abstract

**Introduction:**

HNF4α expression and SMARCA4 loss were thought to be features of non-terminal respiratory unit (TRU)-type lung adenocarcinomas, but their relationships remained unclear.

**Materials and methods:**

HNF4α-positive cases among 241 lung adenocarcinomas were stratified based on TTF-1 and SMARCA4 expressions, histological subtypes, and driver mutations. Immunohistochemical analysis was performed using xenograft tumors of lung adenocarcinoma cell lines with high *HNF4A* expression.

**Result:**

HNF4α-positive adenocarcinomas(*n = *33) were divided into two groups: the variant group(15 mucinous, 2 enteric, and 1 colloid), where SMARCA4 was retained in all cases, and the conventional non-mucinous group(6 papillary, 5 solid, and 4 acinar), where SMARCA4 was lost in 3/15 cases(20%). All variant cases were negative for TTF-1 and showed wild-type *EGFR* and frequent *KRAS* mutations(10/18, 56%). The non-mucinous group was further divided into two groups: TRU-type(*n = *7), which was positive for TTF-1 and showed predominantly papillary histology(6/7, 86%) and *EGFR* mutations(3/7, 43%), and non-TRU-type(*n = *8), which was negative for TTF-1, showed frequent loss of SMARCA4(2/8, 25%) and predominantly solid histology(4/8, 50%), and never harbored *EGFR* mutations. Survival analysis of 230 cases based on histological grading and HNF4α expression revealed that HNF4α-positive poorly differentiated (grade 3) adenocarcinoma showed the worst prognosis. Among 39 cell lines, A549 showed the highest level of *HNF4A*, immunohistochemically HNF4α expression positive and SMARCA4 lost, and exhibited non-mucinous, high-grade morphology in xenograft tumors.

**Conclusion:**

HNF4α-positive non-mucinous adenocarcinomas included TRU-type and non-TRU-type cases; the latter tended to exhibit the high-grade phenotype with frequent loss of SMARCA4, and A549 was a representative cell line.

**Supplementary Information:**

The online version contains supplementary material available at 10.1007/s00428-024-03816-6.

## Introduction

Lung cancer is the leading cause of cancer-related death in many developed countries, including the United States and Japan [1, 2]. Adenocarcinoma is the most common histological subtype of lung cancer [3].

The existence of a distinct subset of lung adenocarcinomas arising from the terminal respiratory unit (TRU) was previously proposed by Yatabe et al. [4–6]. TRU-type lung adenocarcinomas, which are estimated to account for 75%–80% of primary lung adenocarcinomas, show histologically non-mucinous lepidic growth or papillary components and frequently express thyroid transcription factor-1 (TTF-1), which is the master regulator of lung differentiation at high levels [4–6]. The genetic backgrounds of TRU-type adenocarcinomas have been investigated in detail. *Epidermal growth factor receptor* (*EGFR*) mutations and *anaplastic lymphoma kinase* (*ALK*) fusions were found to be specific to TRU-type adenocarcinomas [7, 8]. However, limited information is currently available on non-TRU-type lung adenocarcinomas.

Non-TRU-type lung adenocarcinomas are not a single entity but include various histological and molecular subtypes [9–11]. Kim et al. reported that mucinous adenocarcinomas without TTF-1 expression can be regarded as non-TRU-type lung adenocarcinomas [12], and Yatabe et al. reported that the representative non-TRU-type lung adenocarcinomas were poorly differentiated and exhibited solid morphology [4]. Our previous report revealed that the main group of non-TRU-type lung adenocarcinomas were hepatocyte nuclear factor 4α (HNF4α)-positive adenocarcinomas with gastrointestinal features that frequently harbored *KRAS* mutations and *TTF-1* inactivating mutations/hypermethylation [11].

HNF4α is one of the ligand-dependent transcription factors and specifically expressed in the liver and gastrointestinal organs (stomach, small intestine, and pancreas) but not in normal human lung tissue [13, 14]. HNF4α regulates epithelial cell polarity and morphogenesis and plays an important role in gastrointestinal and hepatic cell differentiation [15–18]. HNF4α also has a role as an oncoprotein and is involved in carcinogenesis, cancer growth, and invasion in various cancers such as hepatocellular cancer, colorectal cancer, gastric cancer, and Barret’s esophageal cancer [19–22]. In the field of lung adenocarcinoma, HNF4α was first reported as a characteristic marker for invasive mucinous adenocarcinomas (IMA) [23], which were regarded as non-TRU-type lung adenocarcinomas. However, the frequency of HNF4α expression in adenocarcinomas other than IMA is not well recognized, especially in non-mucinous lung adenocarcinomas.

SMARCA4 is one of the catalytic subunits in SWI/SNIF chromatin remodeling complexes and has recently been suggested as a tumor suppressor [24–28]. We previously reported that the inactivating mutations of *SMARCA4* were correlated with the epithelial-mesenchymal transition (EMT) phenotype of lung adenocarcinoma cell lines, and loss of SMARCA4 expression was frequent in poorly differentiated non-TRU-type adenocarcinomas, showing a lack of lepidic growth, low expressions of TTF-1 and wild-type *EGFR* [28]. Both the expression of HNF4α and the loss of SMARCA4 are considered characteristics of non-TRU-type adenocarcinomas, but their relationship remains unclear.

This is the first report focusing on the relationships among immunohistochemical expression patterns of HNF4α, TTF-1, and SMARCA4, histological subtypes, and driver mutations. The whole sections of 241 primary lung adenocarcinomas were used in this study. HNF4α expression was found not only in mucinous, enteric, and colloid adenocarcinomas but also in morphologically conventional non-mucinous adenocarcinomas. Some of them heterogeneously expressed HNF4α and TTF-1, which were mutually exclusive within the same tumor. These cases were considered TRU-type adenocarcinomas and frequently harbored *EGFR* mutations. Moreover, TTF-1-negative and HNF4α-positive non-mucinous adenocarcinomas showed wild-type *EGFR* and frequent SMARCA4 loss, and tended to show a high-grade solid morphology and very poor prognosis.

We also examined the histological and immunohistochemical features of xenograft tumors derived from lung adenocarcinoma cell lines. The HNF4α-positive lung adenocarcinoma cell lines (A549, Calu3, H1651, and H2405) all showed non-mucinous and high-grade morphology, and the A549 cell line showed a marked loss of SMARCA4, indicating that it was a representative cell line of HNF4α-positive, non-mucinous lung adenocarcinoma with high-grade morphology.

## Materials and methods

### Case selections

Details are shown in Online Resource 1.

### Histological analysis

Details are shown in Online Resource 1.

### Immunohistochemical analysis

Detailed staining and evaluation protocols are shown in Online Resource 2.

### Sequencing using a next-generation sequencer

Mutations of primary lung tumors were investigated using the MINtS system, employing a MiSeq sequencer (Illumina K.K.), as previously reported [29]. The protocol of RNA extraction is shown in Online Resource 3.

### Cell lines and medium

We used 39 non-squamous non-small-cell lung cancer cell lines. Detailed information is available in our previous reports [9, 28, 30–34].

### Mutational analysis of the 39 cell lines

Gene mutations in the 39 cell lines were based on our previous reports [9, 28, 30–34] and data from the Cancer Cell Line Encyclopedia (https://portals.broadinstitute.org/ccle/).

### Gene expression profile and single nucleotide polymorphism array analyses of 39 lung adenocarcinoma cell lines

A comprehensive gene expression analysis was undertaken using an oligonucleotide microarray (GeneChip Human Genome U133A; Affymetrix), as previously described [35–37]. Analysis with a single nucleotide polymorphism array (Human Mappings 50 K Xbal array; Affymetrix) was performed using the Genome Imbalance Map algorithm, as previously described [38].

### Xenograft tissues of lung adenocarcinoma cell lines

Details are shown in Online resource 4.

### Statistical analysis

For all statistical analyses, SPSS 26 (SPSS, Chicago, IL, USA) was used. Correlations between clinicopathological features and HNF4α expression were analyzed using the χ^2^ test. The Kaplan–Meier method was used for the calculation of survival curves, and the Wilcoxon method was used for comparisons. Multivariate analysis was performed using the Cox proportional hazards model. Differences were considered significant for *p*-values < 0.05.

## Results

### Clinicopathological features of HNF4α-positive adenocarcinomas

**Fig. 1:**
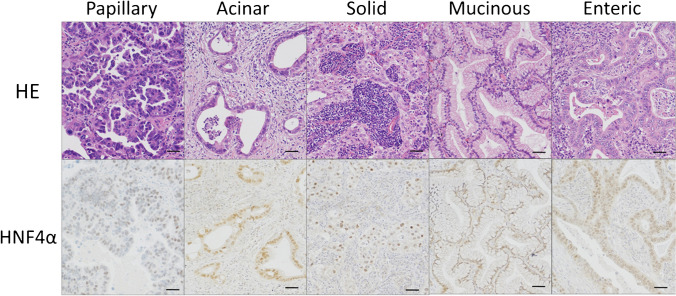
HE (top) and HNF4α staining (bottom) sections from five representative samples of HNF4α-positive lung adenocarcinomas. Scale bar: 50 μm

We conducted an immunohistochemical analysis of HNF4α using 241 primary lung adenocarcinoma samples surgically resected at Jichi Medical University Hospital and found that 33 samples (14%) were positive for HNF4α. Table 1 shows the relationships between HNF4α expression and the clinicopathological features of 238 patients (241 samples). A total of 6 lung adenocarcinoma samples from the 3 patients with double primary lung adenocarcinomas were all positive for TTF-1 and negative for HNF4α. All samples of mucinous (15/15, 100%), enteric (2/2, 100%), and colloid (1/1, 100%) adenocarcinoma exhibited HNF4α expression. HNF4α expression was detected in a proportion of acinar (4/24, 17%), papillary (6/123, 5%), and solid (5/43, 12%) adenocarcinomas. Representative figures of HNF4α-positive lung adenocarcinomas are shown in Fig. 1. None of the HNF4α-positive lung adenocarcinomas showed hepatoid differentiation. None of the in-situ non-mucinous, minimally invasive, or lepidic adenocarcinoma samples (WHO grade 1), representing TRU-type adenocarcinomas, exhibited HNF4α expression.

**Table 1:** Relationships among HNF4α expression and clinicopathologic factors, including expression patterns of TTF-1 and SMARCA4, and genetic status of EGFR, KRAS, ALK, HER2, MET, BRAF, RET, and ROS1 in 241 primary lung adenocarcinomas

	HNF4α expression		
	Positive	Negative	*p-*value ^g^
Age^a^			
60 y/o over 60 y/o less	29 4	170 35	0.476
Sex^a^			
Male Female	21 12	111 94	0.309
Smoking status^b^			
Never Current/Ex-smoker	11 22	87 112	0.263
Hitsology			
AIS/MIA Lepidic adenocarcinoma Papillary adenocarcinoma Acinar adenocarcinoma Micropapillary adenocarcinoma Solid adenocarcinoma Mucinous adenocarcinoma Enteric adenocarcinoma Colloid adenocarcinoma	0 0 6 4 0 5 15 2 1	9 23 117 20 1 38 0 0 0	―
Pathological T stage			
T1 T2-4	6 27	103 105	< 0.001
Pathological Stage^c^			
0-II III-IV	27 6	150 45	0.529
Nodal involvement^c^			
Positive Negative	8 25	56 139	0.631
Pleural invasion			
Positive Negative	15 18	80 128	0.282
Pulmonary metastasis^d^			
Positive Negative	1 31	13 195	0.419
Lymphatic invasion			
Positive Negative	14 19	96 112	0.689
Vessel invasion			
Positive Negative	16 17	100 108	0.556
STAS^e^			
G1-G2 G3	12 6	190 18	0.001
TTF-1			
Positive Negative	7 26	187 21	< 0.001
MUC5AC			
Positive Negative	21 12	11 197	< 0.001
SMARCA4			
Retained Lost	30 3	205 3	0.035
SMARCA2			
Retained Lost	30 3	197 8	0.184
*EGFR* mutation^f^			
Positive Negative	3 30	107 98	< 0.001
*KRAS* mutation^f^			
Positive Negative	13 20	19 186	< 0.001
*ALK* fusion^f^			
Positive Negative	0 33	2 203	0.569
*HER2* mutation^f^			
Positive Negative	0 33	2 203	0.569
*MET* mutation^f^			
Positive Negative	0 33	9 196	0.22
*BRAF* mutation^f^			
Positive Negative	0 33	4 201	0.418
*RET* fusion^f^			
Positive Negative	0 33	2 203	0.569
*ROS1* fusion^f^			
Positive Negative	0 33	3 202	0.484

^a^*n* = 238 because three patients underwent double cancer

^b^*n* = 232 because smoking status was unknown in nine samples

^c^*n* = 228 because we excluded seven samples whose nodal involvement unknown and six double cancer samples

^d^*n* = 240 because pulmonary metastasis in one sample was unknown

^e^*n* = 226 because invasive mucinous adenocarcinomas (*n* = 15) were excluded

^f^*n* = 238 because we were not able to conduct the gene mutation analysis for three samples

^g^Underlined values indicate *p* < 0.05

Table 1 also shows the correlations among HNF4α expression levels and driver mutations, clinicopathological factors and immunohistochemical patterns. In HNF4α-positive samples, the frequency of *KRAS* mutations was significantly high (20/33, 61%) (*p < *0.001), and the frequency of *EGFR* mutations was significantly low (3/33, 9%) (*p < *0.001), whereas no common drive mutations other than *KRAS* and *EGFR* (e.g., *ALK, HER2, MET, BRAF, RET,* or *ROS1*) were found. *EGFR* and *KRAS* mutations were mutually exclusive.

HNF4α expression was correlated with the advanced pT stage (pT2-pT4) (*p = *0.001) and STAS (*p = *0.001), but not correlated with pleural invasion, lymphatic or vessel invasion, intrapulmonary metastasis, or nodal involvement.

Immunohistochemically, HNF4α expression was correlated with a loss of SMARCA4 (*p = *0.035) and MUC5AC expression (*p < *0.001), and inversely correlated with the expression of TTF-1 (*p < *0.001) (Table 1), but seven samples were double-positive for TTF-1 and HNF4α, including six papillary adenocarcinomas and one solid adenocarcinoma. Although the loss of SMARCA2 was not significantly more frequent in HNF4α-positive adenocarcinomas, two of the four HNF4α-positive Grade 3 adenocarcinomas that expressed SMARCA4 showed the loss of SMARCA2.

### TTF-1 and SMARCA4 expression and gene mutation patterns differed in HNF4α-positive lung adenocarcinomas according to histology

**Fig. 2:**
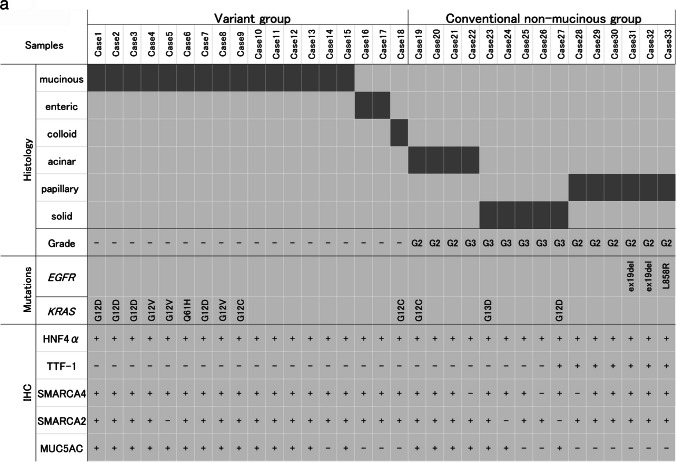

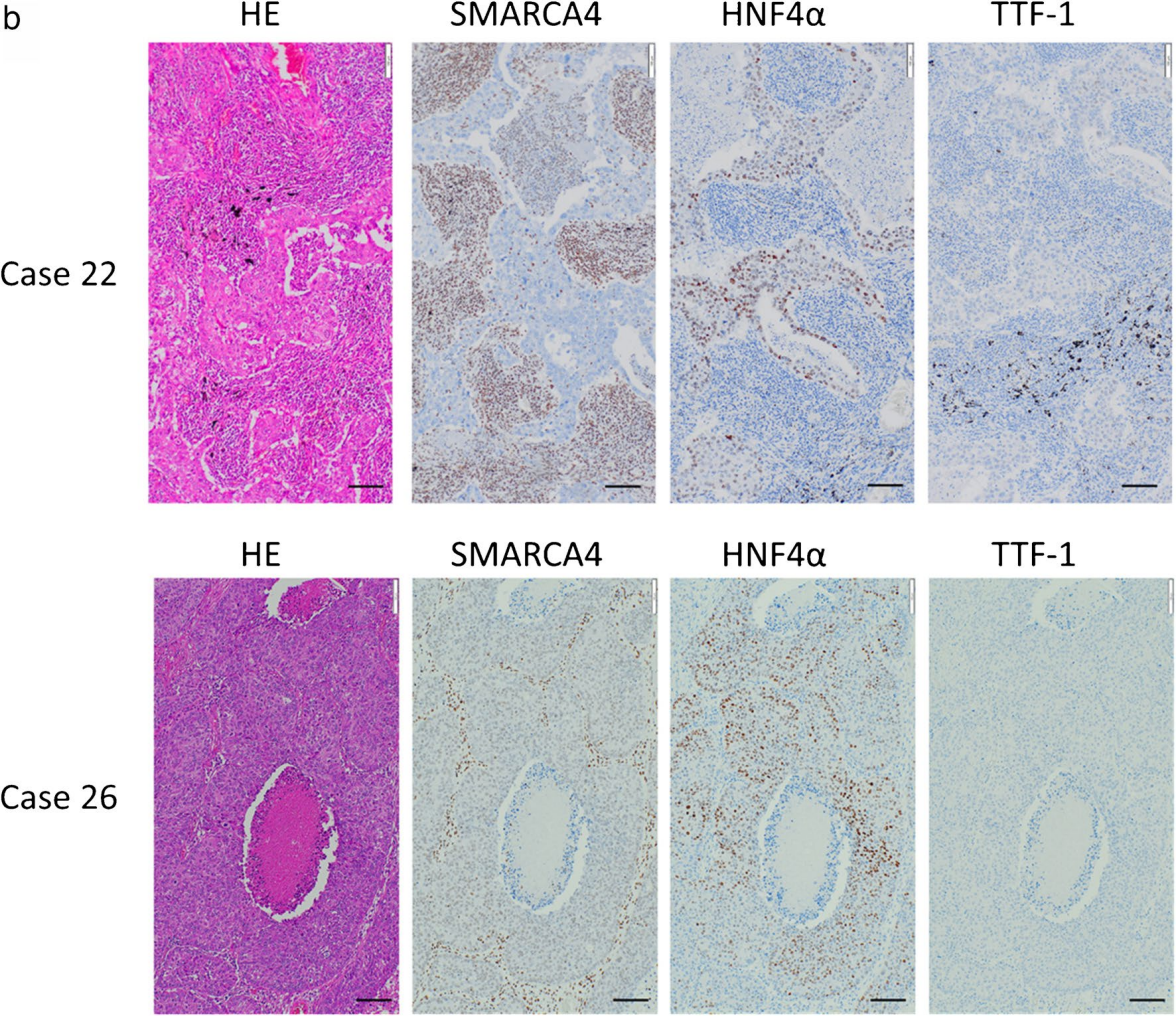
**a** The histological subtypes (mucinous, enteric, colloid, papillary, acinar, and solid adenocarcinomas), histological grades, immunohistochemical expression of HNF4α, TTF-1, SMARCA4, SMARCA2 and MUC5AC and genetic mutations of *EGFR* and *KRAS* in 33 HNF4α-positive lung adenocarcinoma cases, with division into the variant and non-mucinous groups. **b** HE, SMARCA4, HNF4α, and TTF-1 staining of representative cases of HNF4α-positive non-mucinous adenocarcinomas with loss of SMARCA4 (Cases 22 and 26). Both cases were grade 3 adenocarcinomas, SMARCA4 lost, HNF4α-positive, and TTF-1-negative. Note that lymphoid cells within the tumor were SMARCA4-positive (100 × magnification, Scale bar: 100 μm)

Based on the 2021 WHO classification of thoracic tumors [39], we divided HNF4α-positive adenocarcinoma cases (*n = *33) into two groups: the variant group (mucinous, enteric, and colloid adenocarcinomas) (*n = *18) and the conventional non-mucinous group (acinar, papillary, and solid adenocarcinomas) (*n = *15) (Fig. 2a). All variant group cases were diffusely HNF4α-positive and completely TTF-1-negative. None of them harbored *EGFR* mutations, but more than half of the cases harbored the *KRAS* mutation (10/18, 55.6%). In contrast, almost half of the cases in the non-mucinous group were double-positive for TTF-1 and HNF4α (7/15, 46.7%), and their expression patterns were heterogenous and mutually exclusive within the same tumor (Online Resource 5a).

The three *EGFR*-mutated cases in the non-mucinous group were all double-positive for TTF-1 and HNF4α. Given the high frequency of *EGFR* mutations in these double-positive cases (3/7, 43%), we speculated that the double-positive adenocarcinomas were of the TRU-type and that TTF-1-positive TRU-type adenocarcinomas were induced to express HNF4α through the local loss of TTF-1 (e.g., by epigenetic silencing). In addition, all cases in the variant group retained SMARCA4 expression, but in the non-mucinous group, loss of SMARCA4 was detected in 3 of the 15 cases (20%), much more frequently than in HNF4α-negative non-mucinous adenocarcinomas (3/208, 1.4%) (Fig. 2a and Table 1). Figure 2b shows histological images of two representative cases of HNF4α-positive non-mucinous adenocarcinoma with the loss of SMARCA4.

The loss of SMARCA2, a paralog of SMARCA4, did not correlate with the expression of HNF4α (Table 1) and was detected among HNFα-positive cases in both the variant group (5.6%, 1/18) and conventional non-mucinous group (13.3%, 2/15) (Fig. 2a and Online Resource 5b). MUC5AC expression was frequently positive in HNF4α-positive cases (in both the variant and conventional groups), but was almost negative in TTF-1-positive cases (6/7, 85.7%) (Fig. 2a and Online Resource 5b).

### HNF4α-positive non-mucinous adenocarcinomas with high-grade morphology (WHO grade 3) showed the worst prognosis

**Fig. 3:**
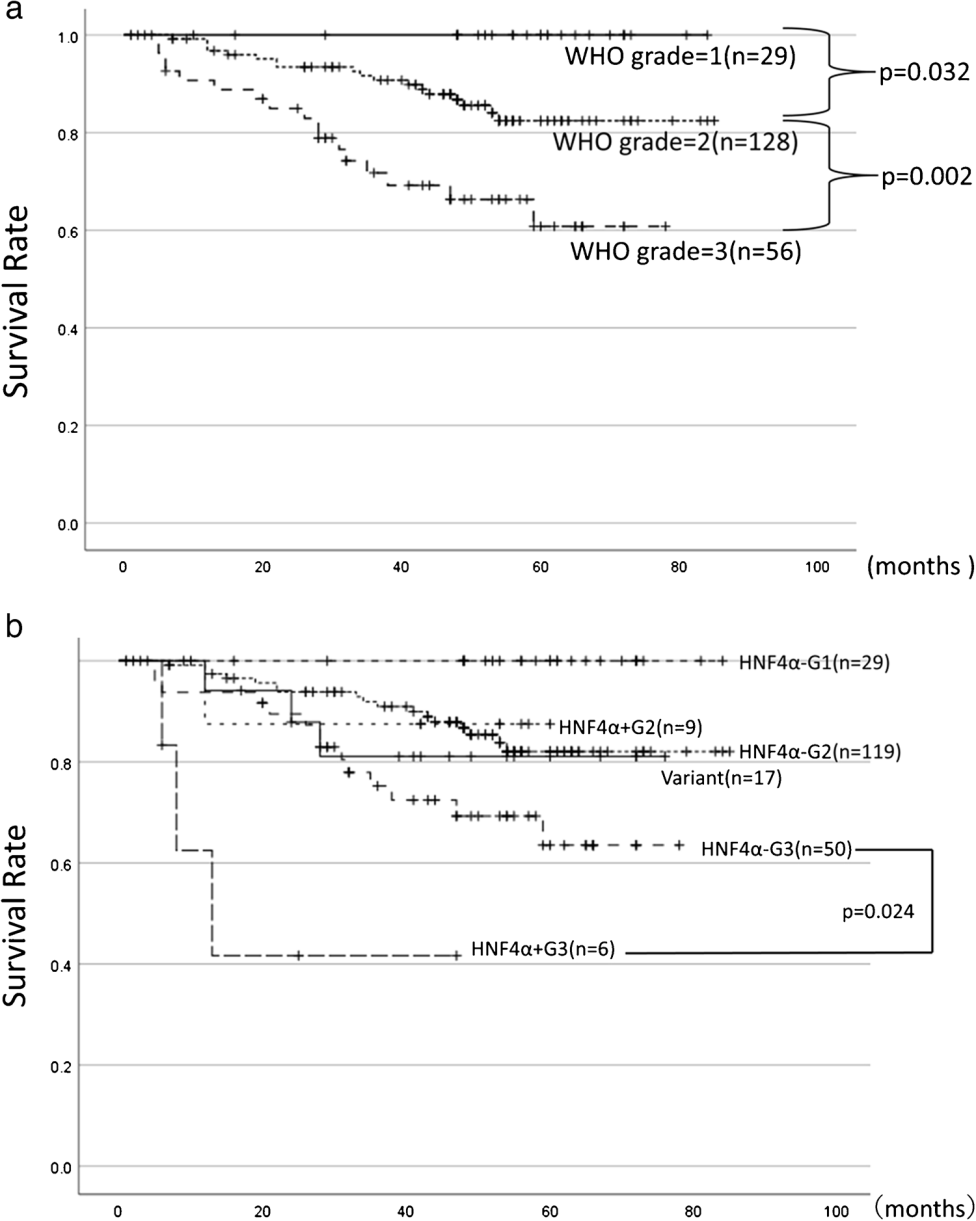
**a** Overall survival among 213 cases of non-mucinous adenocarcinomas categorized according to the WHO grading system. **b** The prognoses of 230 lung adenocarcinomas were analyzed in 6 groups; HNF4α + G3: HNF4α-positive grade 3 (*n = *6), HNF4α-G3: HNF4α-negative grade 3 (*n = *50), HNF4α + G2: HNF4α-positive grade 2 (*n = *9), HNF4α-G2: HNF4α-negative grade 2 (*n = *119), HNF4α-G1:HNF4α-negative grade 1 (*n = *29), and the variant group (*n = *17). The samples with unknown prognoses (*n = *5) and double carcinoma cases (*n = *3) were excluded

The three-tiered grading system is the common prognostic indicator of non-mucinous lung adenocarcinomas [39]. In the present study, the 5-year survival rates of grade 1 (*n = *29), grade 2 (*n = *128), and grade 3 (*n = *56) groups were 100%, 86.0%, and 61.4% respectively, and the survival rates differed significantly (grade 1 vs. grade 2: *p = *0.032, grade 2 vs. grade 3: *p = *0.002) (Fig. 3a). Next, for survival analysis, we re-classified non-mucinous adenocarcinoma cases of each grade group into HNF4α-positive and HNF4α-negative groups: HNF4α-positive grade 3 group (*n = *6), HNF4α-negative grade 3 group (*n = *50), HNF4α-positive grade 2 group (*n = *9), HNF4α-negative grade 2 group (*n = *119), and HNF4α-negative grade 1 group (*n = *29), as well as the variant group (*n = *17). Notably, the HNF4α-positive grade 3 group showed worse prognosis than the HNF4α-negative grade 3 group (3-year survival rates of 51.4% and 69.3%, respectively) (*p = *0.024), showing the worst prognosis among the six groups (Fig. 3b).

We found that in grade 3 non-mucinous adenocarcinomas (*n = *56), sex, pleural invasion, pStage, HNF4α expression and MUC5AC expressions, were poor prognostic factors (Online Resource 6a). We performed a multivariate analysis, excluding the expression of MUC5AC, which correlated with the expression of HNF4α, and found that the expression of HNF4α and the pStage remained significant in the multivariate analysis (HR, 3.318; CI, 1.344–8.188 for HNF4α expression and HR, 9.019; CI, 4.107–19.804 for pStage) (Online Resource 6b). Although HNF4α-positive grade 3 non-mucinous adenocarcinomas frequently showed the loss of SMARCA4 (2/6, 33%), it was not identified as a poor prognostic factor (Online Resource 6a).

We also compared clinicopathological factors among the six groups (Online Resource 7) and found that advanced pT factor, advanced pStage, lymph node metastasis, vessel invasion, pleural invasion, and pulmonary metastasis were most frequently observed in the HNF4α-positive grade 3 group, indicating that this group was the aggressive phenotype.

### Xenograft tumors of HNF4α-positive lung adenocarcinoma cell lines showed high-grade, non-mucinous morphology

Finally, we examined whether HNF4α-positive grade 3 adenocarcinoma cell lines were present among the 39 non-squamous non-small cell lung cancer cell lines. Online Resource 8 shows the gene-level expressions of *HNF4A* and *TTF-1* in the 39 cell lines. The four cell lines with the highest expression of *HNF4A* were A549, H2405, Calu-3, and H1651, in that order. Online Resource 8 also shows the common driver mutations of the 39 cell lines, and among the four *HNF4A*-high cell lines, *SMARCA4* and *KRAS* mutations were found in A549, *HER2* amplification was found in Calu-3, and no common driver mutations were found in H2405 or H1651.

**Fig. 4:**
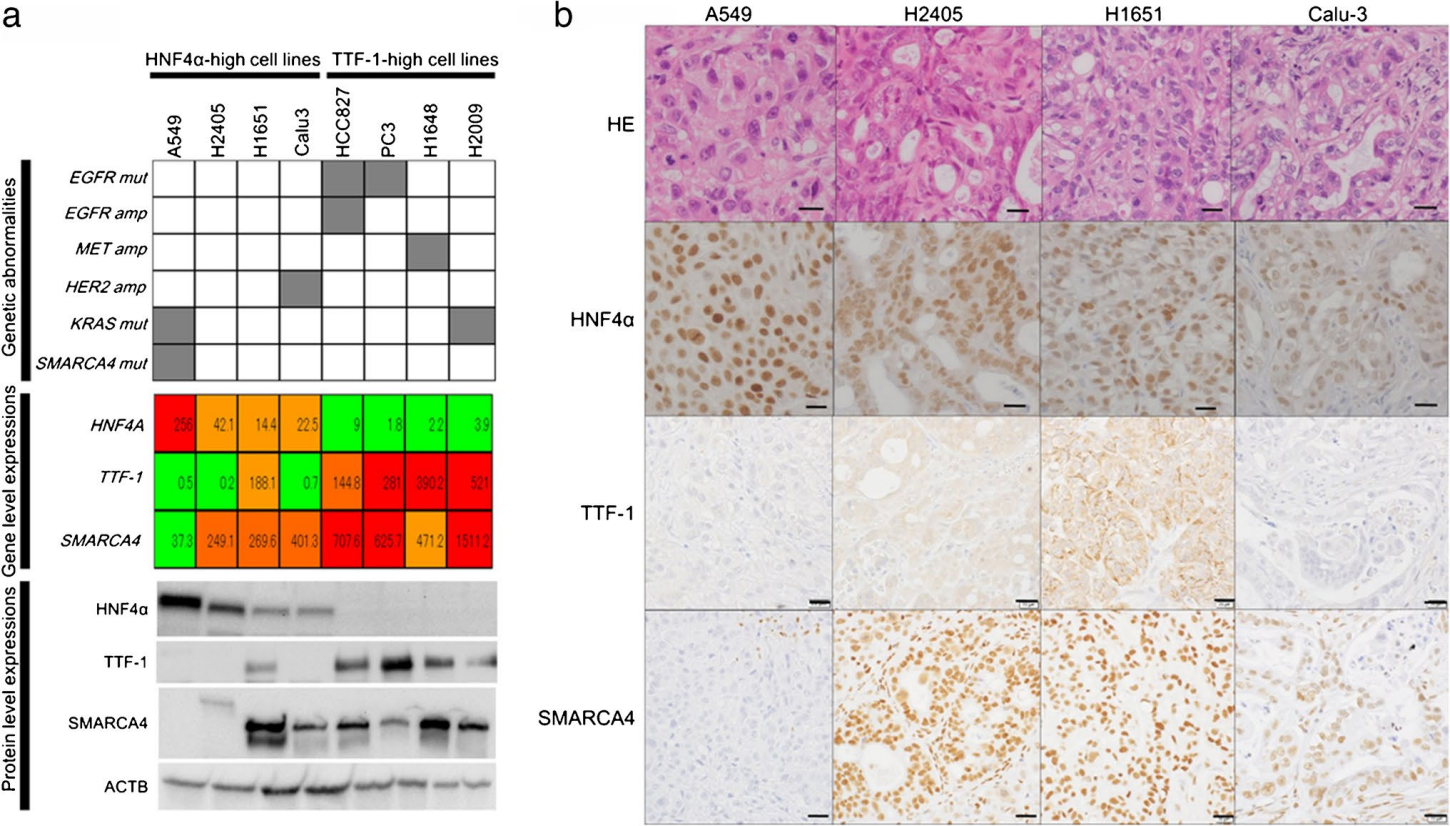
**a** Genetic status of *EGFR*, *MET*, *HER2*, *KRAS*, and *SMARCA4* (upper panel), gene-level expressions of *HNF4A*, *TTF-1*, and *SMARCA4* (middle panel), and protein expression levels of HNF4α, TTF-1, SMARCA4, and ACTB (lower panel) for 8 cell lines, including the four cell lines that highly express HNF4A (A549, H2405, H1651, and Calu-3) and the four cell lines that highly express TTF-1 (HCC827, PC3, H1648, and H2009). In the upper panel, the gray box indicates the presence of genetic abnormalities and the white box indicates the absence of genetic abnormalities. In the middle lane, red means more than or equal to the average of each gene expression, orange means under the average but more than or equal to one-quarter of the average, and green means under one-quarter of the average. **b** The histological features and immunohistochemical expression patterns of HNF4α, TTF-1, and SMARCA4 for the xenograft tumors of A549, H2405, H1651, and Calu-3

Figure 4a summarizes (i) the genetic status of *EGFR, MET, HER2, KRAS,* and *SMARCA4* (upper panel), (ii) gene-level expressions of *HNF4A, TTF-1,* and *SMARCA4* (middle panel), and (iii) protein-level expressions of HNF4α, TTF-1, SMARCA4, and ACTB (lower panel) for the four *HNF4A*-high cell lines (A549, H2405, H1651, and Calu3), compared with the four representative TRU-type cell lines with *TTF-1*-high expressions (HCC827, PC3, H1648, and H2009). The four *HNF4A*-high cell lines showed high HNF4α expression and low level of TTF-1, except for H1651, at both the gene and protein levels. A marked decrease in the expression level of SMARCA4 was only observed in *SMARCA4*-mutated A549, whereas the other three *HNF4A*-high cell lines exhibited SMARCA4 expression. An aberrant band of SMARCA4 was detected in H2405 by western blot analysis (Fig. 4a).

Next, using xenograft tumors of the four *HNF4A*-high cell lines, we examined the histological growth patterns in HE staining and performed immunohistochemical analysis for HNF4α, TTF-1, and SMARCA4 (Fig. 4b). A549 and H1651 showed solid growth patterns, H2405 showed solid growth patterns with focal cribriform patterns, and Calu-3 showed fused glandular and papillary growth patterns (Fig. 4b, the top row). All of these growth patterns are features of grade 3 primary lung adenocarcinoma, and notably, none of the cell lines showed morphological features of mucinous adenocarcinoma. Immunohistochemically, all of the four *HNF4A*-high cell lines were HNF4α-positive and TTF-1-negative in the nucleus (Fig. 4b, the second and third row), but H1651, which showed high TTF-1 expression at both the gene and protein levels, exhibited intracytoplasmic TTF-1 expression. SMARCA4 expression was diffusely lost in the A549 xenograft tumor but retained in the other three cell lines (Fig. 4b, the bottom row).

## Discussion

Here, we have shown that HNF4α expression was not limited to mucinous, enteric, or colloid adenocarcinomas, which showed gastrointestinal morphology, but also appeared in morphologically conventional non-mucinous adenocarcinomas such as acinar, papillary, and solid adenocarcinomas.

In the present study (mostly Asian cases), the frequency of *KRAS* mutations was significantly higher in HNF4α-positive adenocarcinomas (39.4%, 13/33 cases) than in HNF4α-negative adenocarcinomas (9.2%, 19/207 cases). Based on The Cancer Genome Atlas data of 456 primary lung adenocarcinomas (mostly Caucasian cases), the frequency of *KRAS* mutations was not significantly higher in *HNF4A*-high cases (35.9%, 42/117 cases) than in *HNF4A-low* cases (29.5%, 100/339 cases) (*p = *0.121) (Online resource 9). *KRAS* mutations in lung adenocarcinoma are more frequent in Caucasians than in Asians. We speculate that this is not because of the higher frequency of HNF4α-positive cases in Caucasians, but because of the higher frequency of *KRAS* mutations in HNF4α-negative adenocarcinomas (mainly TRU-type lung adenocarcinomas) in Caucasians.

The results obtained herein revealed the absence of common driver mutations other than *KRAS* mutations in mucinous adenocarcinomas (60%, 9/15 cases). *NRG1* gene fusion, which has been reported in *KRAS* wild-type mucinous adenocarcinomas, was not examined in the present study [40]. *CD74*-*NRG1* fusion genes have been identified as driver oncogenes and *ERBB2*/*ERBB3* receptors may be the target of these fusion genes [41]. We previously noted that the knockdown of *HNF4A* in lung adenocarcinoma cell lines suppressed the expression and phosphorylation of *ERBB3* (data not shown). The relationship among *ERBB3*, *HNF4A*, and *NRG1* fusion genes is a topic for future studies. Although they were not identified in the present study, *ALK* fusion genes have been detected in mucinous adenocarcinomas [42], but are TTF-1-positive TRU-type adenocarcinomas with a mucinous morphology, a distinct entity from HNF4α-positive mucinous lung adenocarcinomas, exhibiting gastrointestinal features.

In the present study, the frequency of TTF-1 expression, loss of SMARCA4, and *EGFR* mutations differed according to histology. All cases of mucinous, enteric, and colloid adenocarcinoma were HNF4α-positive, TTF-1-negative, and SMARCA4 retained, and showed a high frequency of *KRAS* mutations (10/18, 55.6%) and no *EGFR* mutations (0/18, 0%). A recent study showed that 16 cases of enteric and mucinous adenocarcinoma lacked common driver mutations except for *KRAS* mutations [43], indicating that mucinous, enteric, and colloid adenocarcinomas might form a single spectrum of HNF4α-positive non-TRU-type adenocarcinomas showing gastrointestinal differentiation. However, enteric adenocarcinomas occasionally show focal TTF-1 expression and *EGFR* mutations [44], suggesting that some enteric adenocarcinomas may be phenotypically altered from TRU-type adenocarcinomas. The etiology of enteric adenocarcinoma is controversial and requires further investigation.

Approximately half of the HNF4α-positive non-mucinous adenocarcinomas were TTF-1-positive (7/15, 47%), and they frequently showing papillary predominant histology (6/7, 86%). They were often accompanied by a non-mucinous lepidic component (5/7, 71%) and often lacked MUC5AC expression (1/7, 14.3%). Furthermore, approximately half of the cases harbored *EGFR* mutations (3/7, 43%), suggesting that the double-positive cases for HNF4α and TTF-1 were derived from TRU-type lung adenocarcinomas. In these cases, HNF4α and TTF-1 were expressed heterogeneously and were mutually exclusive within the same tumor. We speculate that focal loss of TTF-1 expression may be partially due to *TTF-1* gene hypermethylation, as previously reported [11].

The remaining half of the HNF4α-positive non-mucinous adenocarcinomas were totally TTF-1-negative (8/8, 100%) and never harbored *EGFR* mutations (0/8, 0%), suggesting that they were the non-TRU-type lung adenocarcinomas. Half of them were poorly differentiated solid adenocarcinomas (4/8, 50%), potentially differing from mucinous, enteric, and colloid adenocarcinomas, which showed histologically gastrointestinal differentiation. We previously reported that HNF4α was not a significant prognostic factor in lung adenocarcinomas at any stage [10], but confirmed that the expression of HNF4α and a solid morphology were independent poor prognostic factors in advanced stage samples. In this study, HNF4α-positive poorly differentiated (grade 3) non-mucinous adenocarcinomas were aggressive phenotypes and showed the worst prognosis, and HNF4α expression was an independent prognostic factor in grade 3 non-mucinous lung adenocarcinomas. These results suggest that the expression of HNF4α plays a distinctive role in the progression of lung adenocarcinoma and a poor prognosis.

HNF4α was recently shown to be involved in the growth and invasion of various cancers as oncoprotein [19–22]. A previous study that examined the expression of HNF4α and mucin profiles in lung mucinous adenocarcinomas [45] reported that HNF4α induced the expression of MUC3 in *KRAS*-mutated mucinous adenocarcinomas, which is a poor prognostic factor for mucinous adenocarcinomas of the breast and appendix [46, 47]. Chen et al. demonstrated that the HNF4α-BC200-FMR-positive feedback loop promoted cell growth and metastasis in *KRAS*-mutated, HNF4α-positive cell lines (A549) [48]. Therefore, the expression of HNF4α has potential as a therapeutic target in lung adenocarcinomas, particularly *KRAS*-mutated lung adenocarcinomas.

Herein, we found that HNF4α-positive non-mucinous adenocarcinomas frequently showed loss of SMARCA4 (3/15, 20%), much more frequently than in mucinous, enteric, and colloid adenocarcinomas (0/18, 0%), and HNF4α-negative non-mucinous adenocarcinomas (3/208, 1.4%). Additionally, loss of SMARCA4 was more frequently observed in HNF4α-positive grade 3 adenocarcinomas (2/6, 33%). The function of SMARCA4 varies among different organs and diseases, and SMARCA4 inactivation in lung cancer is related to the loss of lung lineage transcription and early metastasis [49]. We speculated the HNF4α expression in grade 3 adenocarcinoma may imply dedifferentiation associated with the inactivated SMARCA4 function, resulting in high-grade morphology and poor prognosis.

In the present study, loss of SMARCA4 was only found in 2.5% (6/241) of the lung adenocarcinoma samples. *SMARCA4* mutation rates were reported to account for approximately 8% of non-small cell lung cancers, but not all mutations resulted in loss of SMARCA4 expression [49]. Some variants of *SMARCA4* mutation may show intact SMARCA4 expression despite the loss of its function [50]. Note that, unlike HNF4α, loss of SMARCA4 was not an independent prognostic factor in grade 3 adenocarcinomas in our study (Online Resource 6), but we did not investigate the mutational status of *SMARCA4* in SMARCA4-retained adenocarcinomas. Further studies are needed to elucidate the relationship between HNF4α expression and the function of SMARCA4.

We also demonstrated that four lung adenocarcinoma cell lines (A549, H1651, H2405, and Calu-3) had high HNF4α expression at both the gene and protein levels. All four cell lines tended to show high expression levels of *Vimentin* and *ZEB1* compared with the *TTF-1-*high cell lines, and relatively low expression of *CDH1* (Online Resource 10), indicating dedifferentiation or EMT. All four cell lines may be regarded as representatives of non-mucinous HNF4α-positive lung adenocarcinomas with grade 3 morphology, but their immunohistochemical and genetic features varied. We propose that A549 is not a mucinous adenocarcinoma cell line [48], but A549 may be a representative cell line of HNF4α-positive grade 3 lung adenocarcinomas with aggressive pathological features.

In conclusion, a subset of HNF4α-positive adenocarcinomas, such as mucinous adenocarcinomas with gastrointestinal differentiation, are TTF-1-negative and SMARCA4 retained, often showing *KRAS* mutations. In addition, some conventional non-mucinous adenocarcinomas are HNF4α-positive, which include not only TRU-type adenocarcinomas that are double-positive for TTF-1 and HNF4α but also non-TRU-type poorly differentiated (grade 3) adenocarcinomas with frequent loss of SMARCA4 expression. HNF4α-positive grade 3 adenocarcinoma shows a very poor prognosis, and HNF4α expression is an independent prognostic factor in grade 3 lung adenocarcinomas. Thus, examining the status of HNF4α expression is important for not only assuming the etiology and gene mutational status but also predicting the prognosis in non-mucinous adenocarcinomas. The A549 cell line may be considered a representative cell line of HNF4α-positive grade 3 adenocarcinomas.

## Supplementary Information

Below is the link to the electronic supplementary material.

Supplementary file1 (DOCX 7992 KB)

## Data Availability

Raw data can be obtained from the corresponding author upon reasonable request.

## Code availability

N/A.
