# Supplementary material for: Expression patterns of HNF4α, TTF-1, and SMARCA4 in lung adenocarcinomas: impacts on clinicopathological and genetic features
Source: Virchows Arch. 2024 May 6;486(2):343–54. doi: 10.1007/s00428-024-03816-6 (PMC11876232; doi:10.1007/s00428-024-03816-6)
Supplement: Supplementary file 1 — Supplementary file1 (DOCX 7992 KB) [file 428_2024_3816_MOESM1_ESM.docx]

**Supplementary Information (Online resources 1-10)**

**Article title:** Expression patterns of HNF4α, TTF-1, and SMARCA4 in lung adenocarcinomas: Impacts on clinicopathological and genetic features

**Journal name:** Virchow Archiv

**Author names:** Hitomi Kawai^1,2^, Tamaki Miura^3^, Natsumi Kawamatsu^2^, Tomoki Nakagawa^2^, Aya Shiba-Ishii^1^, Taichiro Yoshimoto^4^, Yusuke Amano^3^, Atsushi Kihara^3^, Yuji Sakuma^5^, Kazutaka Fujita^6^, Tomoki Shibano^7^, Shumpei Ishikawa^8^, Tetsuo Ushiku^9^, Masashi Fukayama^9^, Hiroyoshi Tsubochi^7^, Shunsuke Endo^7^, Koichi Hagiwara^10^, Daisuke Matsubara^1,2,3^, Toshiro Niki^3^

**Affiliation:** 1 Department of Pathology, University of Tsukuba, 1-1-1 Tennodai, Tsukuba, Ibaraki, 305-8574, Japan

2 Department of Diagnostic Pathology, University of Tsukuba Hospital, 2-1-1- Amakubo, Ibaraki, 305-8576, Japan

3 Department of Integrative Pathology, Jichi Medical University, 3311-1, Yakushiji, Shimotsuke, Tochigi, 329-0498, Japan

4 Department of Pathology, Showa General Hospital, 8-1-1 Hanakoganei, Kodaira-shi, Tokyo, 187-851, Japan

5 Department of Molecular medicine, Sapporo Medical University, 1-17, minami, chuoku, Sapporo, Hokkaido, 060-8556, Japan

6 Department of Respiratory Medicine, Jichi Medical University, 3311-1, Yakushiji, Shimotsukeshi, Tochigi, 329-0498, Japan

7 Department of Thoracic Surgery, Jichi Medical University, 3311-1, Yakushiji, Shimotsukeshi, Tochigi, 329-0498, Japan

8 Department of Preventive Medicine, Graduate School of Medicine, the University of Tokyo, 7-3-1, Hongo, Bunkyo-ku, Tokyo, 113-0033, Japan

9 Human Pathology Department, Graduate School of Medicine, the University of Tokyo, 7-3-1, Hongo, Bunkyo-ku, Tokyo, 113-0033, Japan

10 Omiya Medical Association Medical Examination Center, 2-107, Higashioonari-chou, Kita-ku, Saitama-shi, Saitama 331-8689, Japan

**E-mail address of the corresponding author:** matsubarad@md.tsukuba.ac.jp **(**Daisuke Matsubara, M.D., Ph.D.)

**Online resource 1: Detailed information about patients enrolled and histological analysis in this study**

A total of 238 patients who underwent surgical resection at Jichi Medical University Hospital between 2010 and 2013 were enrolled in this study. Three of the 238 patients had double primary lung adenocarcinoma, so 241 samples were used in the analysis. The patients included 132 men and 106 women, ranging in age between 36 and 84 years (average, 67.6 years). Three patients experienced simultaneous lung adenocarcinomas, and thus 241 surgical specimens of lung adenocarcinoma were obtained. Seven samples were not examined for nodal metastasis. Three samples were not suitable for gene analysis because of an insufficient amount of material for the analysis. Five samples were lost for follow-up, and the prognosis was unknown. Informed consent was obtained from all patients, and the study was approved by the Institutional Ethics Review Committee.

Two pathologists (HK and TM) independently reviewed each sample. Histological diagnosis and grading were determined according to the criteria outlined in the WHO Classification of Tumours (5th edition, Thoracic Tumours) [1]. TNM classification and pathological stage were reassigned based on the International Association for the Study of Lung Cancer 8th edition lung cancer staging system [2].

In non-mucinous adenocarcinomas, the predominant subtype and WHO grade (grades 1–3: well to poorly differentiated) were determined based on only the histological features of hematoxylin and eosin (HE) specimens, not considering the immunohistochemical features. Adenocarcinomas with mucinous features, which showed intracytoplasmic and/or extracytoplasmic mucin, were diagnosed as mucinous adenocarcinoma. IMA was included in mucinous adenocarcinomas. Spread through air spaces (STAS) was graded into 3 grades as described in our previous report [3].

**Online resource 2: Detailed information about the protocol of immunohistochemistry**

Formalin-fixed, paraffin-embedded tumor specimens were analyzed by immunohistochemistry for the expression of TTF-1, HNF4α, SMARCA4, SMARCA2 and MUC5AC. Tissue sections were treated with 0.3% hydrogen peroxide in methanol for 30 minutes to block endogenous peroxidase activity and then autoclaved in 10 mmol/L citrate buffer (pH 6.0) at 120°C for 10 minutes for TTF-1, HNF4α and SMARCA4, in 10 mmol/L citrate buffer (pH 7.0) at 120℃ for 10 minutes for SMARCA2, and in 10 mmol/L citrate buffer (pH 6.0) at 95℃ for 10 min in a microwave oven for MUC5AC. Sections were then preincubated with 10% normal horse serum in PBS, incubated with a mouse monoclonal anti-human TTF-1 Ab (M3575, clone 8G7G3/1) from Dako at a dilution of 1:100, a mouse monoclonal anti-human HNF4α (H1415) Ab from Perseus Proteomics at a dilution of 1:60, and a rabbit monoclonal anti-human SMARCA4 (ab110641, clone EPNCIR111A) at a dilution of 1:200, a rabbit monoclonal anti-human BRM Ab (D9E8B) from Cell signaling at a dilution of 1:600, and a mouse monoclonal anti-human MUC5AC Ab (NCL-MUC-5AC) from Leica Biosystems at a dilution of 1:100, at 4°C overnight. All Abs were detected using a streptavidin-HRP conjugate according to the manufacturer’s instructions. 3,3′-Diaminobenzidine tetrahydrochloride was used as a chromogen, and hematoxylin was used as a light counterstain. When staining was undertaken on cancer tissue sections, a positive control section was also stained, and we always confirmed that positive control cells were correctly stained. Immunohistochemical staining was independently evaluated by 2 pathologists (HK and TM). Immunoreactivity was scored based on the percentage of cells that stained positively. The expression of TTF-1, MUC5AC and HNF4α in a tumor was defined as positive when at least 5% of tumor cells were stained and negative when less than 5% were stained. Loss of SMARCA4/SMARCA2 (SMARCA4/SMARCA2 lost) was defined when more than 10% of tumor cells showed the loss of SMARCA4/SMARCA2 expression. SMARCA4/SMARCA2 retained was defined when 100% tumor cells were positive for SMARCA4.

**Online resource 3: Detailed information about the protocol of RNA extraction**

For all samples in this study, RNA was extracted from frozen tumor samples for analysis using the MINtS system. Add 500 µL of TRIZOL to a sampling tube (1.5 mL) containing 3–4 mm-sized tissue. Grind the tissue, and when it is fine, add 500 µL of TRIZOL and mix well. Next, add 200 µL of chloroform and vortex for 15 seconds. After centrifugation at 15,000 rpm for 15 min at 4°C, take the supernatant and mix by inversion with 500 µL of isopropyl alcohol. Incubate at room temperature for 10 min, then centrifuge at 12,000 rpm for 10 min at 4°C. Take and discard the supernatant so as not to absorb the pellet at the bottom of the tube and add 1 mL of 75% ethanol and mix invertedly. After centrifugation at 4°C for 5 min at 7,500 rpm, discard the supernatant, taking care not to absorb the pellet, and dry the pellet at room temperature. Dissolve in 50 µL of dH_2_O (autoclave). Measure absorbance and store at −80°C.

**Online resource 4: Detailed information about the protocol of Xenograft tissues of lung adenocarcinoma cell lines**

We established xenograft tumors of the lung cancer cell lines by injecting cell suspensions (5 × 106) into the flanks of 6-week-old female severe combined immunodeficient mice NOD C.B-17-Prkdc scid/J (NOD/SCID). From the 39 cell lines, we successfully established 27 xenograft tumors. Mice were sacrificed after six weeks or were immediately sacrificed when weight loss of 20% or more occurred or the maximum tumor size was larger than 10 mm, to prevent suffering. Mice were deeply anesthetized by inhalation of 4% isoflurane and euthanized by manual cervical dislocation. Subcutaneous tumors were harvested, fixed with 10% formalin, and embedded in paraffin blocks. Tumor histology was examined after HE staining.

**Online resource 5:**

**5a: HE (a), TTF-1 (b), and HNF4α (c) staining on serial sections of the representative adenocarcinoma showing double positivity for TTF-1 and HNF4α**


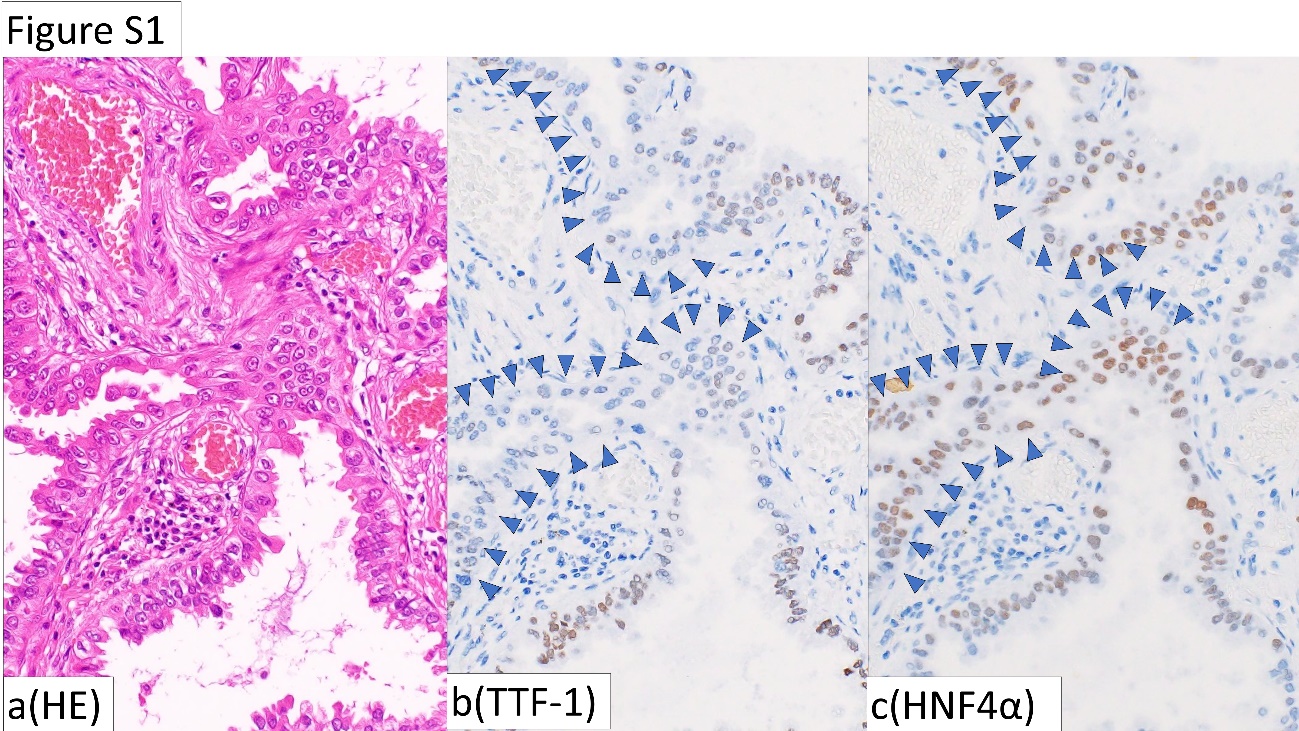


Arrow heads indicate TTF-1-negative (b) and HNF4α-positive (c) cells

5b: Representative images of adenocarcinomas showing SMARCA2 lost (a: case number 24, b: case number 27) and MUC5AC (c: case number 19, d: case number 20).


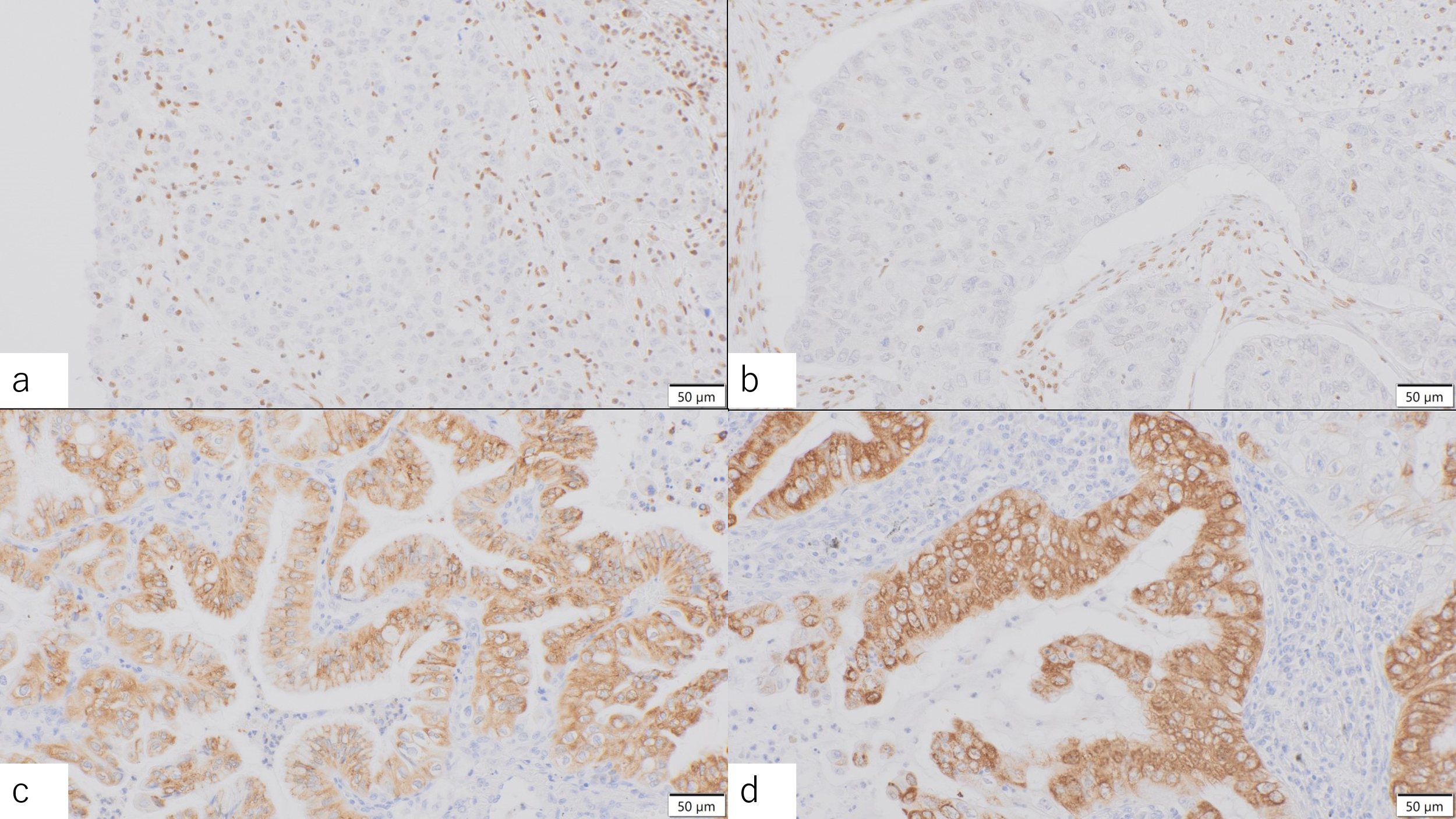


**Online resource 6: The results of univariate (6a) and multivariate (6b) Cox regression survival analyses**

| 6a: Univariate Cox regression analysis, G3 (n = 56) | | |  |
| --- | --- | --- | --- |
| Pathological feature | Hazard ratio | 95% Confidence interval | p-value |
| HNF4a (negative vs positive) | 3.958 | 1.084–14.450 | 0.037 |
| MUC5AC (negative vs positive) | 9.438 | 2.641-33.729 | <0.001 |
| Age ( < 60 vs. ≥ 60) | 4.087 | 0.535–31.217 | 0.175 |
| Sex (male vs. female) | 0.186 | 0.042–0.815 | 0.026 |
| SMARCA4 (retain vs. loss) | 0.846 | 0.112–6.422 | 0.872 |
| SMARCA2 (retain vs. loss) | 0.876 | 0.199-3.852 | 0.861 |
| TTF-1 (negative vs. positive) | 0.606 | 0.233–1.571 | 0.303 |
| pT(pT1 vs. pT2-4) | 2.415 | 0.916–6.369 | 0.075 |
| pl (pl0 vs. pl1-3) | 2.952 | 1.035–8.417 | 0.043 |
| pm (pm0 vs. pm1-3) | 1.223 | 0.279–5.363 | 0.789 |
| Ly (Ly0 vs. Ly1) | 2.859 | 0.653–12.508 | 0.163 |
| V (V0 vs. V1) | 2.47 | 0.564–10.807 | 0.23 |
| pN (pN0 vs. pN1-3) | 2.518 | 0.970–6.537 | 0.058 |
| pStage (pStage I-II vs. III-IV) | 3.923 | 1.485–10.365 | 0.006 |

| 6b: Multivariate Cox regression analysis, G3 (n = 56) | | | |
| --- | --- | --- | --- |
| Pathological feature | Hazard ratio | 95% Confidence interval | p-value |
| HNF4a (negative vs. positive) | 3.318 | 1.344–8.188 | 0.009 |
| Sex (male vs. female) | 0.595 | 0.297–1.194 | 0.144 |
| pl (pl0 vs. pl1-3) | 1.776 | 0.920–3.429 | 0.087 |
| pStage (pStage I-II vs. III-IV) | 9.019 | 4.107–19.804 | < 0.001 |

**Online resource 7: The clinicopathological factors among 6 groups, namely HNF4α-positive grade 3 (HNF4α+G3), HNF4α-negative grade 3 (HNF4α-G3), HNF4α-positive grade 2 (HNF4α+G2), HNF4α-negative grade 2 (HNF4α-G2), HNF4α-negative grade 1 (HNF4α-G1), and the mucinous, enteric, and colloid adenocarcinoma group (mucinous, etc.), according to the three-tiered grading system proposed by the IASLC Pathology Committee [1] and the expression of HNF4α**

|  | HNF4α+G3, n (%) | HNF4α-G3, n (%) | HNF4α+G2, n (%) | HNF4α-G2, n (%) | HNF4α-G1, n (%) | Mucinous, etc., n (%) |
| --- | --- | --- | --- | --- | --- | --- |
| Age ^a^  60 y/o, over  60 y/o, below | 5 (83.3)  1 (16.7) | 46 (86.8)  7 (13.2) | 8 (88.9)  1 (11.1) | 102 (82.9)  21 (17.1) | 22 (75.9)  7 (24.1) | 16 (88.9)  2 (11.1) |
| Sex ^a^  Male  Female | 6 (100)  0 (0) | 32 (60.4)  21 (39.6) | 4 (44.4)  5 (55.6) | 66 (53.7)  57 (46.3) | 13 (44.8)  16 (55.2) | 11 (63.6)  7 (36.4) |
| Pathological T stage  T1  T2-4 | 0 (0)  6 (100) | 21 (39.6)  32 (60.4) | 1 (11.1)  8 (87.9) | 56 (44.4)  70 (55.6) | 26 (89.7)  3 (10.3) | 5 (38.5)  13 (61.5) |
| Pathological stage ^b^  0-II  III-IV | 3 (50.0)  3 (50.0) | 36 (70.6)  15 (49.4) | 8 (88.9)  1 (11.1) | 87 (75.0)  29 (25.0) | 27 (96.4)  1 (3.6) | 16 (88.9)  2 (11.1) |
| Nodal involvement ^b^  Positive  Negative | 3 (50.0)  3 (50.0) | 16 (31.8)  35 (68.2) | 1 (11.1)  8 (88.9) | 38 (32.8)  78 (67.2) | 2 (7.1)  26 (92.9) | 4 (28.6)  14 (71.4) |
| Pleural invasion  Positive  Negative | 4 (66.7)  2 (33.3) | 26 (49.1)  27 (50.9) | 5 (55.6)  4 (44.4) | 51 (40.5)  75 (59.5) | 3 (10.3)  26 (89.7) | 6 (33.3)  12 (66.7) |
| Pulmonary metastasis ^c^  Positive  Negative | 1 (16.7)  5 (83.3) | 5 (9.4)  48 (90.6) | 0 (0)  8 (100) | 8 (6.3)  118 (93.7) | 0 (0)  29 (100) | 0 (0)  18 (100) |
| Lymphatic invasion  Positive  Negative | 4 (66.7)  2 (33.3) | 41 (77.4)  12 (22.6) | 3 (33.3)  6 (66.7) | 49 (38.9)  77 (61.1) | 5 (17.2)  24 (82.8) | 7 (36.4)  11 (63.6) |
| Vessel invasion  Positive  Negative | 6 (100)  0 (0) | 41 (77.4)  12 (22.6) | 6 (66.7)  3 (33.3) | 54 (42.9)  72 (57.1) | 5 (17.2)  24 (82.8) | 4 (28.6)  14 (71.4) |
| STAS ^d^  G1-G2  G3 | 5 (83.3)  1 (16.7) | 48 (90.1)  5 (9.9) | 4 (44.4)  5 (55.6) | 113 (89.7)  13 (10.3) | 29 (56.3)  0 (43.7) | 3 (100)  0 (0) |
| TTF-1  Positive  Negative | 1 (16.7)  5 (83.3) | 33 (62.3)  20 (37.7) | 6 (66.7)  3 (33.3) | 125 (99.2)  1 (0.8) | 29 (100)  0 (0) | 0 (0)  18 (100) |
| MUC5AC  Positive  Negative | 4 (66.7)  2 (33.3) | 2 (3.8)  51 (96.2) | 3 (33.3)  6 (66.6) | 9 (7.1)  117 (92.9) | 0 (0)  29 (100) | 14 (71.4)  4 (28.6) |
| SMARCA4  Retained  Lost | 4 (66.7)  2 (33.3) | 51 (96.2)  2 (3.8) | 8 (89.9)  1 (11.1) | 125 (99.2)  1 (0.8) | 29 (100)  0 (0) | 18 (100)  0 (0) |
| SMARCA2  Retained  Loss | 4 (66.7)  2 (33.3) | 48 (90.6%)  5 (9.4%) | 9 (100%)  0 (0%) | 123 (97.6)  3 (2.4) | 29 (100)  0 (0) | 17 (94.4%)  1 (5.6%) |

a) n = 238 because three patients underwent double cancer.

b) n = 228 because we excluded seven samples whose nodal involvement was unknown and six double cancer samples.

c) n = 240 because pulmonary metastasis in one sample was unknown.

d) Invasive mucinous adenocarcinomas (n = 15) were excluded.

**Online resource 8: The presence of genetic abnormalities for *EGFR, MET, HER2, ALK, RET, KRAS, BRAF*, and *SMARCA4* and the gene-level expressions of *CDH1, Vimentin, ZEB1, HNF4A, TTF-1*, and *SMARCA4* in 39 cell lines; if xenograft tumors have been successfully established, their histological features are also shown**

#: Red means above the average value and green means below the average value.

*: The gray box indicates the presence of a mutation (mut), amplification (amp), or fusion, the white box indicates wild-type or silent mutations, and the yellow box indicates unknown. Data were revised from our previous report [4].**Online resource 9: The relationship between *HNF4A* expression level and *KRAS* mutation based on TCGA data of 456 lung adenocarcinomas samples, which were thought to be composed if mostly Caucasians (https://portal.gdc.cancer.gov/projects/TCGA-LUAD). The cutoff of *HNF4A* expression was 5. We defined HNF4A gene expression levels greater than 5 as high expression because 5 was less than the first quantile of the *HNF4A* gene expression levels in mucinous adenocarcinomas and greater than the third quantile of the *HNF4A* gene expression level of non-mucinous adenocarcinoma.**

|  | *HNF4A* | | p-value |
| --- | --- | --- | --- |
| *KRAS* | High, n (%) | Low, n (%) |  |
| Mutant | 42 (35.9) | 100 (29.5) | 0.121 |
| wildtype | 75 (64.1) | 239 (70.5) |  |

**Online resource 10: Gene-level expressions of *vimentin* (a), *ZEB1* (b), and *CDH1* (c) for *HNF4A*-high level cell lines (A549, H2405, H1651, and Calu3) and *HNF4A*-low level cell lines (HCC827, PC3, H1658, and H2009)**


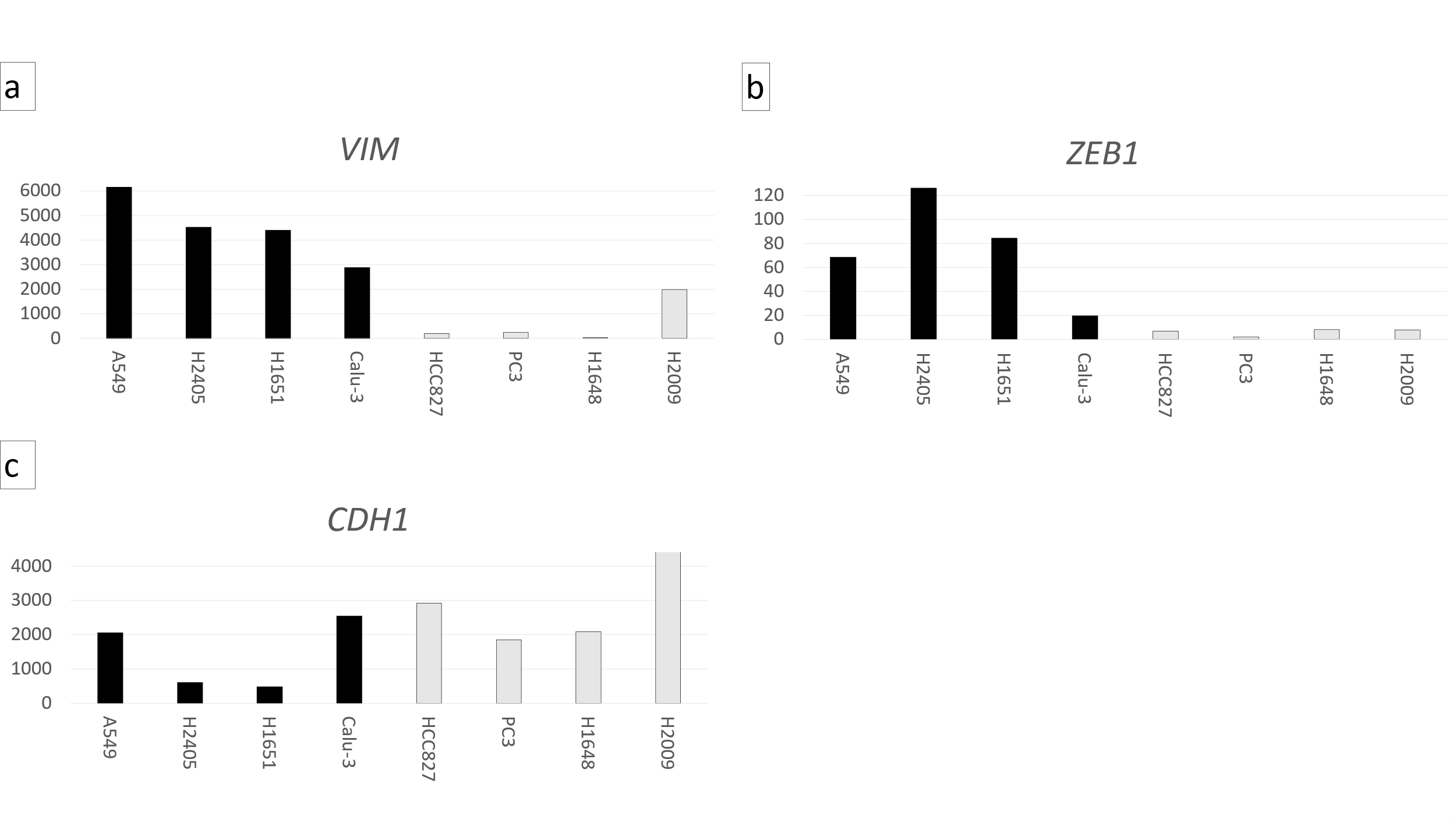


Reference

1. WHO Classification of Tumours Editorial Board. Thoracic Tumours. Thoracic Tumours. 5th ed. Lyon, France: International Agency for Research on Cancer; 2021.

2. Detterbeck FC, Boffa DJ, Kim AW et Tanoue LT. The eighth edition lung cancer stage classification. Chest. 2017; 151(1): 193-203. doi: 10.1016/j.chest.2016.10.010.

3. Matsubara D, Yoshimoto T, Soda M et al. Reciprocal expression of trefoil factor-1 and thyroid transcription factor-1 in lung adenocarcinomas. Cancer sci. 2020; 111(6): 2183-2195. doi: 10.1111/cas.14403.

4. Matsubara D, Yoshimoto T, Akolekar N et al. Genetic and phenotypic determinants of morphologies in 3D cultures and xenografts of lung tumor cell lines. Cancer Sci. 2023; 114(4): 1757-1770. doi: 10.1111/cas.15702.
